# 1763. A study on the effectiveness of a pharmacist led Antifungal stewardship program, in immunocompromised patients of a tertiary care teaching hospital in South-India.

**DOI:** 10.1093/ofid/ofac492.1393

**Published:** 2022-12-15

**Authors:** Priscilla Rupali, Jisha Sara John, Amita Jacob, Rajiv Karthik, Hanna Alexander, Sushil Selvarajan, Biju George

**Affiliations:** Christian Medical College, Vellore, Tamil Nadu, India; Christian Medical College, Vellore, Vellore, Tamil Nadu, India; Christian Medical College, Vellore, Vellore, Tamil Nadu, India; Christian Medical College, Vellore, Vellore, Tamil Nadu, India; Christian Medical College, Vellore, Tamil Nadu, India; Christian Medical College, Vellore, Vellore, Tamil Nadu, India; CHristian Medical College, Vellore, Vellore, Tamil Nadu, India

## Abstract

**Background:**

Anti-fungal stewardship (AFS) is a less explored area of an anti-microbial stewardship (AMS) program as the patients prone to fungal infections are mostly immunocompromised, post-chemotherapy or post-transplant. In a Low-and- Middle income country (LMIC) like India, there is a dearth of Infectious Disease (ID) trained physicians and pharmacists. We aimed to study the effectiveness of a pharmacist led AFS program to ensure rational prescribing of antifungals via a post-prescription review and feedback method.

**Methods:**

In this prospective interrupted time series analysis from June 2021 to November 2021, AFS was done on adult in-patients in the department of Hematology in a tertiary care teaching hospital in South India. The study had a pre-intervention phase and intervention phase of 3 months each. In the pre-intervention phase, patients on anti-fungal therapy > 48 hours were identified and base line data were collected and no recommendations were given. In the intervention phase, in those on antifungals >48 hours, appropriate recommendations were made with regard to modification and discontinuation of the anti-fungals based on patients’ clinical condition under the supervision of an ID physician. Acceptance and impact of the intervention were monitored and recorded.

METHOD OF THE STUDY

**Figure:**
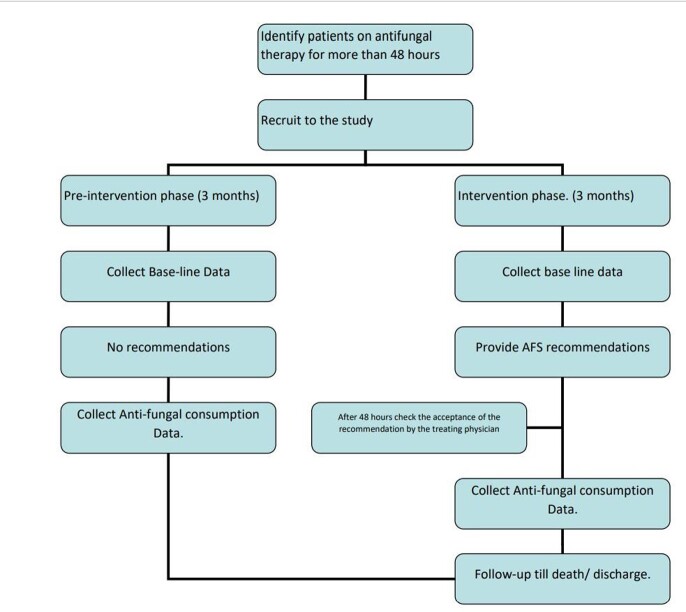

The study was a prospective study with 2 phases : pre-intervention and intervention. In the intervention phase, the appropriateness of the Anti-fungal therapy was analyzed and recommendations were given.

**Results:**

A total of 193 patients were analyzed over 6 months, of which 107 patients with a mean age of 42.1 ± 14.2 belonged to the pre-intervention phase and 86 patients aged 40.2 ±12.6 years were in the intervention phase. There was no statistically significant difference in the in-hospital mortality [26.16% vs 23. 25% (p = 0.64)] between the two groups. In the intervention phase, 15 (17.44%) prescriptions were found to be inappropriate. Among these 66% of the recommendations were accepted by the treating physician. The days of therapy per 100 patient days were calculated for each individual anti-fungal drug and there was a significant reduction in consumption of Anidulafungin [29.648 Vs 14.28 (p < 0.0007)], Amphotericin B [42.05 Vs 22.18 (p< 0.0001)] and Voriconazole [56.41 Vs 35.77 (p< 0.00001)] in the intervention phase.

**Figure:**
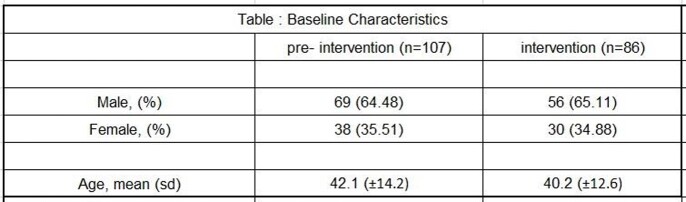

Outcome measurements

**Figure:**
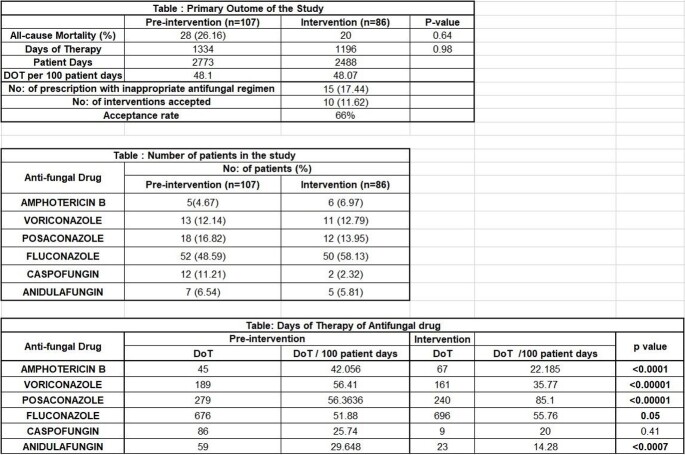

**Figure:**
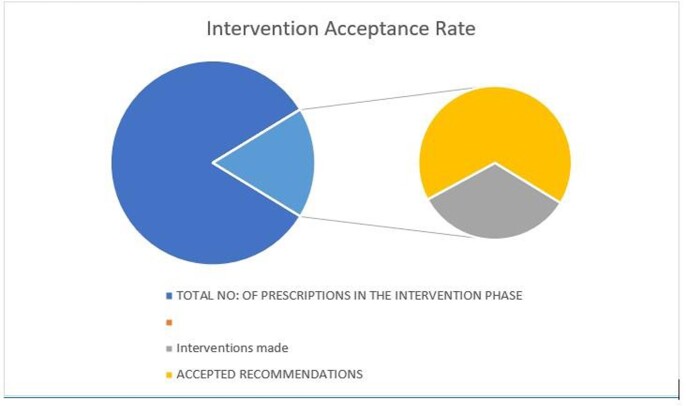

**Conclusion:**

A pharmacist led AFS program resulted in statistically significant reduction in the consumption of antifungals, without a significant difference in the in-hospital mortality.

**Disclosures:**

**Priscilla Rupali, MD, DTM&H**, PFIZER: Grant/Research Support|PFIZER: Grant/Research Support.

